# MRI for radiotherapy guidelines: A comparative review of recommendations by IPEM topical report, AAPM TG‐284, and NCS report 36

**DOI:** 10.1002/mp.70529

**Published:** 2026-07-16

**Authors:** Rob. H. N Tijssen, Steven. F Petit, Mariska de Smet, Zdenko van Kesteren, Carri Glide‐Hurst, Eric Paulson, Richard Speight, Michael. J Dubec, Ellen. J. L Brunenberg, Joost. P. A Kuijer

**Affiliations:** ^1^ Department of Radiation Oncology Catharina Hospital Eindhoven Eindhoven, North Brabant The Netherlands; ^2^ Department of Biomedical Engineering Technical University Eindhoven Eindhoven The Netherlands; ^3^ Department of Radiation Oncology Erasmus MC Cancer Institute Rotterdam The Netherlands; ^4^ Department of Radiotherapy Amsterdam University Medical Center Amsterdam The Netherlands; ^5^ Department of Human Oncology and Medical Physics University of Wisconsin‐Madison Madison Wisconsin USA; ^6^ Department of Radiation Oncology Medical College of Wisconsin Milwaukee Wisconsin USA; ^7^ Leeds Cancer Centre Leeds Teaching Hospitals NHS Trust Leeds UK; ^8^ Christie Medical Physics and Engineering The Christie NHS Foundation Trust Manchester UK; ^9^ Department of Radiation Oncology Radboud University Medical Center Nijmegen The Netherlands; ^10^ Department of Radiology and Nuclear Medicine Amsterdam University Medical Center Amsterdam The Netherlands

**Keywords:** guidelines, MR‐Linac, quality assurance

## Abstract

In recent years, three international guidelines on the use of MRI in radiotherapy (RT) have been published, each offering valuable recommendations. However, the coexistence of multiple guidelines can make it challenging for practitioners to determine which to follow in specific clinical contexts. This review aims to clarify where the guidelines converge, where they differ, and how they can be used together to support informed decision‐making. Authored by contributors from the AAPM (US), IPEM (UK), and NCS (NL) this overview provides a concise yet comprehensive comparison of their scope, content, and recommendations. This synthesis serves as a practical and informative resource for the medical physics community, supporting more consistent and informed implementation of MRI in RT.

## 1 INTRODUCTION

**Computed** tomography (CT) imaging has been the primary image modality to delineate tumour and organ‐at‐risk structures for treatment planning in radiotherapy (RT). However, due to its superior soft tissue contrast compared to CT, magnetic resonance imaging (MRI) has become increasingly common in RT since its introduction to RT over two decades ago.^1^, ^2^ Initially, MRI was used mainly for radiotherapy treatment planning (RTP),^3^ but its role has expanded in recent years to include onboard MRI guidance systems.^4^, ^5^

Despite this growing adoption, comprehensive guidelines on the clinical implementation of MRI in RT have been lacking until recently. Between 2021 and 2023, three major medical physics societies released guidelines on the implementation, the use, and quality assurance (QA) of MRI in RT:

1. Topical report: *guidance on the use of MRI for external beam RT treatment planning* by the Institute of Physics and Engineering in Medicine.^6^
2. Task Group 284 report: *MRI simulation in RT; implementation, optimisation, and QA* by the American Association of Physicists in Medicine.^7^
3. Report 36: *QA of MRI for RT* by the Netherlands Commission on Radiation Dosimetry.^8^

This editorial compares these three current guidelines, highlights the similarities, and differences to assist readers in selecting the most relevant source(s) for their specific needs.

### 1.1 IPEM topical report

The Institute of Physics and Engineering in Medicine (IPEM) has a strong history of publishing internationally adopted guidelines. In 2016, a lack of guidance on MRI in RT was identified at an IPEM MRI Guided RT meeting. To address this, an IPEM working party was formed, reviewing literature and gathering consensus from UK users. The group produced three key publications: two highlighting variations in practice across the UK and internationally,^3^, ^9^ and one providing guidelines.^6^ These guidelines, developed by a multidisciplinary team, have been endorsed by IPEM, the Society and College of Radiographers (SCoR), and the Faculty of Clinical Oncology of The Royal College of Radiologists (RCR), ensuring a unified approach to implementing MRI in RT. These guidelines focused on those setting up and using a dedicated MR scanner for RT and who have limited time on an MR scanner.

### 1.2 AAPM TG 284

AAPM Task Group 284 entitled “MRI Simulation in RT: Considerations for Clinical Implementation, Optimization, and QA”, was published in 2021.^7^ The report describes the use and implementation of dedicated and dual‐purpose magnetic resonance simulation (MR‐sim) platforms in radiation oncology. TG‐284 provides recommendations for equipment selection, system siting, and routine QA tests, including tolerances. Strategies for optimizing clinical workflows, imaging of patients in the treatment position and guidance on patient‐specific considerations such as managing motion and distortions are shared. The report includes recommendations for MRI safety protocols in hybrid environments including guidance on training, operational models, and staffing levels for safe implementation of MR‐sim. Examples of optimized scanning protocols and templated checklists for QA programs are included.

### 1.3 NCS report 36

The NCS is the Netherlands Commission on Radiation Dosimetry (*Nederlandse Commissie voor Stralingsdosimetrie*) and was established in 1982 with the mission of promoting the appropriate use of ionizing radiation dosimetry for scientific research and practical applications. Since then, the NCS has regularly published reports providing recommendations for professionals, primarily in the fields of nuclear medicine, radiology and radiation therapy. NCS reports are primarily written in English and freely accessible (www.radiationdosimetry.org), making them widely referenced internationally.

In 2017, Subcommittee 36 was formed to develop guidelines for the QA of MRI in RT. The resulting report focuses extensively on system QA and quality control (QC) across various RT applications of MRI, ranging from the use of diagnostic MRI to onboard MRI‐guided linear accelerators (MR‐Linacs).^8^

## 2 CONTENT OVERVIEW

All three guidelines were developed to provide direction on the implementation and use of MRI in RT. A comparison of their focus areas and content is presented in Table 1. At first glance, there is a significant overlap between the guidelines, which is reassuring, as it confirms that experts from different regions agree on the key aspects of MRI integration in RT. However, differences are also present due to variations in scope and areas of special focus, making the guidelines complementary to each other rather than simply overlapping.

**TABLE 1:** Content overview of each guideline.

	IPEM Topical Report	AAPM TG‐284	NCS Report 36
**General**			
Title	IPEM topical report: guidance on the use of MRI for external beam radiotherapy treatment planning	MRI simulation in radiotherapy; implementation, optimisation, and quality assurance	Quality assurance of MRI for radiotherapy
Publication date	Feb 2021	July 2021	March 2023
Main focus area	Implementation and QA of MRI simulation	Implementation and QA of MRI simulation	QA programme, incl. detailed instructions per test
MRI use cases (applications) covered	MR‐sim for RTP (co‐registered to CT)	Conventional MR‐sim for RTP (co‐registered to CT) Inverted workflow (MR‐sim prior to CT‐sim)	Diagnostic MRI (side‐by‐side to CT) MR‐sim (co‐registered to CT) MR‐only (no CT) MR‐guided (MR‐Linac)
**Specific content**			
Purchase and installation	Establishing system requirements Site planning specifications Project management (stakeholders)	Establishing system requirements Site planning specifications	–
Clinical deployment and application	Staffing & Training Collaboration between RD and RT MR‐RT workflow (incl. patient setup) Protocol optimisation for RT Motion management Post Processing	Staffing & Training Collaboration between RD and RT MR‐RT workflow (incl. patient setup) Protocol optimisation for RT Motion management Post Processing	Basic MR physics (background for RT physicist) Workflow per MR use case described Protocol optimisation for RT Challenges and recommendations per use case
MR safety	Standard of practice (ref. MHRA ^10^) Training requirements Required Documentation MR safety labels Equipment checks	Standard of practice (ref. ACR ^11^,^12^) Training requirements Responsibility allocation Patient screening checklist MR safety labels Equipment checks Contrast agents	Standard of practice (ref. ACR^11^,^12^ and NVKF ^13^) Training requirements Responsibility allocation Patient screening MR safety labels Equipment checks
Quality Assurance	Overview of tests for commissioning and periodic QA Tolerances and frequencies	Overview of tests for commissioning and periodic QA Tolerances and frequencies	Detailed description of tests for commissioning and periodic QA Tolerances and frequencies Appendix with imaging QA instructions (how‐to)
**Unique features**	Recommendations on MRI to CT registration	MRI safety checklist / Example FMEA of clinical workflow Disease site‐specific MR simulation setups	Distinction between the various MR‐RT applications

The IPEM and AAPM reports, both published in 2021, are the most similar in terms of content. Both guidelines focus on MR‐sim for RT treatment planning, offering recommendations on site planning, installation, staffing, training, collaboration with radiology departments, patient workflows, and protocol optimization. While both follow a similar structure, each report includes unique topics—for example, the IPEM report provides recommendations on image registration. The AAPM report includes template checklists for periodic and patient QA, as well as reference MR simulator scan protocols.

The NCS guideline, published in 2023, takes a broader approach to complement the existing IPEM and AAPM guidelines. Instead of focusing solely on MRI simulation, it also covers diagnostic MRI, MR‐only workflows, and MR‐Linac applications. The NCS report has a strong focus on system QA. Unlike the IPEM and AAPM reports, which provide recommendations on the introduction of MRI into a RT department, the NCS report starts with a basic MR physics section, which allows RT physicists with limited MRI experience to gain the foundational knowledge to understand the rationale behind the described QA tests. The appendix provides step‐by‐step instructions for performing these tests, including phantom recommendations.

Regarding MR Safety, all three reports offer similar recommendations on organisational setup, safety zoning and screening procedures. However, each report references its respective local codes of practice and legislation for specific details—for example on exposure limits and risk assessment requirements.

## 3 COMPARING THE RECOMMENDED QA & QC TESTS

The recommended system QA and QC tests represent vital checks that are part of the overall quality management program.^14^ When establishing a QA/QC protocol, the Medical Physics Expert, MR Safety Expert, and other responsible staff now have access to three different guidelines. It is essential to consider whether the QA/QC protocols proposed by these guidelines align with each other, overlap, or can be used as complementary resources. To address this question, we will review the tests outlined in each of the three guidelines, comparing their associated tolerances and recommended frequency. We will evaluate whether the recommended QA/QC protocols are consistent across all three guidelines and provide an explanation for any observed differences.

Table 2 presents an overview of the recommended tests as described in the three guidelines. For brevity, optional tests are excluded, and only critical (action level) tolerances are listed below.

**TABLE 2:** Detailed overview of the recommended tests in each guideline.

		IPEM Topical Report (application: MR‐sim)		AAPM TG‐284 (application: MR‐sim)		NCS Report 36 (applications: 1. diag. MRI, 2. MR‐sim, 3. MR‐only, 4. MR‐Linac)		
	Parameter	Tolerances	frequency	tolerances	frequency	tolerances	frequency	Application
**1**	**SNR**	Reference to ^17^, ^18^, ^19^	C, E, A	action limit +/‐ one standard deviation from baseline	C, E, A	combined image: 20% (crit) from baseline uncombined image: 30% from baseline	C, E, M	1,2,3,4
**2**	**Image uniformity**	Reference to ^17^, ^18^, ^19^	C, E, A	action limit +/‐ one standard deviation from baseline	C, E, A	6% from baseline	C, E	1,2,3,4
**3**	**Ghosting**	Reference to ^17^, ^18^, ^19^	C, E, A	2.50%	M	3%	C, E	1,2,3,4
**4**	**Image artefacts**	Reference to^17^, ^18^, ^19^	C, E, A	no obvious artefacts	M	no artefacts present	C, E	1,2,3,4
**5**	**Gradient fidelity (linear and/or non‐linear components)**	2 mm within relevant volume, or 1 mm for SRS. At acceptance: note volume where displacement > 1 mm and > 2 mm.	C, E, A*	< 1 mm for 10 cm radius, < 2 mm for 20 cm radius	C, E*	small FoV phantom: < 1.5% or < 1 mm (SRS) Large phantom: < 1 mm for 10 cm radius, < 2 mm for 17.5 cm radius, < 2.5 mm for 20 cm radius	C, E*	1,2,3,4
**6**	**Resonance frequency**	–	–	< 1 ppm/day during acceptance testing < 0.25 ppm/day first 1–2 months operation	C, E	0.1% per month	C, E	1,2,3,4
**7**	**RF transmit amplitude**	–	–	vendor specified	C, E, A	30% from baseline	C, E	1,2,3,4
**8**	**Shim (B_0_ homogeneity)**	2 mm, or 1 mm for SRS within relevant volume	C, E	< 0.5 ppm VRMS across 35 cm DSV or vendor specified	C, E, A	application specific. MR‐sim: < 2 ppm peak‐to‐peak for 35 cm DSV MR‐Linac: < 128 Hz peak‐to‐peak for 25 cm DSV	C, E, Q	1,2,3,4
**9**	**Couch positioning**	0.2 deg laterally, 0.5 deg long	C, E, A	C,E: 0.3 deg between couch and MR C,E: < 0.5 mm discrepancy in vertical offset M: 2 mm	C, E, M	0.3 deg between couch and MR 1 mm discrepancy in vertical offset	C, E, Q/M	−,2,3,4
**10**	**Connectivity and orientation**	1–2 mm	C, E	site specific per TG‐248 guidelines	C, E	Correct in DICOM header and TPS	C, E	−,2,3,4
**11**	**Motion verification**	–	–	2 mm amplitude deviation compared to waveform	C, E	–	–	–
**12**	**External laser position**	+/− 2 mm	C, E, M	C,E Isocentricity: < 1 mm M Isocentricity: < 2 mm	C, E, A	Orthogonality: 0.5 deg, Vertical position: 1 mm, Isocentricity: 2 mm	C, E, M	−,‐,3,4*
**13**	**Synthetic CT generation**	–	–	–	–	*no strict tolerances set, only preferred limits*	C, E	−,‐,3,‐
**14**	**End‐to‐End test**	< 2 mm for most RT applications	–	Data integrity via TG53. Concordance landmarks < 2 mm. Co‐reg max error 0.5 voxel size	C, E	Equal to local tolerances for CT‐sim	C, E	‐,‐,3,‐
**15**	**MR‐MV coincidence**	–	–	–	–	< 1 mm	C, E, M	‐,‐,‐,4
**16**	**B_0_ direction**	–	–	–	–	in agreement with B_0_ direction in TPS	C, E	‐,‐,‐,4
**17**	**Gantry angle dependent B_0_ homogeneity & MR isocenter shift**	–	–	–	–	shim peak‐to‐peak: < 128 Hz apparent image shift: < 1 mm	C, E, A	‐,‐,‐,4
**18**	**Linac induced RF interference**	–	–	–	–	Pass/Fail	C, E, A	‐,‐,‐,4
**19**	**Radiation induced artefacts**	–	–	–	–	Pass/Fail	C, E, A	‐,‐,‐,4
**20**	**Temporal stability test**	–	–	–	–	< 1 mm image drift	C, E, A	‐,‐,‐,4
**21**	**Real‐time feedback latency**	–	–	–	–	< 100 ms deviation from baseline	C, E, A	‐,‐,‐,4

*Note*: Suggested frequency is indicated by C, commissioning/acceptance; E, equipment change; A, annually; M, monthly. The last column refers only to the NCS report and indicates which application each test applies to: 1. diagnostic MRI, 2. MR‐sim, 3. MR‐only, 4. MR‐Linac.

Abbreviations: DSV, diameter spherical volume; FoV, field‐of‐view; SRS, stereotactic radiosurgery; TPS, treatment planning system; VRMS, volume root‐mean‐square;

^*^ minimum frequency is annual or after equipment change, but more frequent checks are recommended to test stability over time.

When reviewing the types of tests recommended, we observe a high level of agreement between the reports. The majority of the tests (1–14) are included in all three reports, but some differences are noted:

‐ Test 6 and Test 7, concerning resonance frequency (f_0_) and RF transmit amplitude, are not included in the IPEM report. These tests were not explicitly included as f_0_ and RF transmit amplitude were considered to be stable on modern systems, and significant fluctuations would likely be detected by other tests including B_0_ field mapping and signal‐to‐noise (SNR) assessment.
‐ Test 11, related to motion verification, is a specialty topic included only in the AAPM report. This is due to the fact that margin definition based on dynamic MRI is a more recent development and is not yet widely used. However, MR based motion assessment is gaining traction and a dedicated AAPM report on 4D‐MRI is being prepared by Task Group No. 391^15^ at the time of writing.
‐ Test 13, on synthetic CT, is also marked as a specialty topic and is only covered by the NCS report. Like 4D‐MRI, synthetic CT (MR‐only) is still an emerging field that is still under development. Consequently, it is likely that test procedures and corresponding tolerances will evolve in the future. Users are therefore advised to consult the latest scientific literature to stay informed about recent advances and potential challenges in synthetic CT methods.
‐ Test 12, concerning external laser position, illustrates a more nuanced difference. While both the IPEM and AAPM report recommends periodic checks for MR‐sim and specifies strict tolerances, the NCS states that the required accuracy depends on the specific application. For traditional MR‐sim (MRI registered to CT) no strict tolerances are specified, while for MR‐only, the tolerances are aligned with those of CT‐simulators.
‐ Tests 15–21 focus on MR‐Linac systems, a specialty topic covered only by the NCS report as guidance from AAPM is forthcoming from TG‐352 “MR‐guided RT Systems: Considerations for clinical implementation and QA” .^16^

With respect to the tolerances and frequency of the QC tests, some differences are observed for the more generic image quality tests (e.g., Tests 1–4, 7, 8). For these tests, all reports rely on the existing guidelines established in radiology. The AAPM and NCS reports both list tolerances, but they are selected from different existing guidelines, which is why the units that are used to express the tolerances in (e.g., percentage vs. standard deviation or ppm), are different. The IPEM directs the reader to the existing guidelines, without providing a selected set of tolerances.

For the QC checks that directly influence the accuracy of the RT treatment chain, however, (e.g., Tests 5,8–14), all reports provide an extensive list of tolerances that are specific to RT that, when stated, exhibit a high level of agreement.

For these tests we observe only subtle differences:

‐ When reviewing test 5, gradient fidelity, it is important to note that two sources can contribute to gradient errors: 1) errors in gradient amplitude calibration, which results in a global scaling error (i.e., the linear component), and 2) errors that produce local geometric offsets (i.e., the gradient non‐linearity, which is inherent to the design of the gradient, is therefore constant over time, and typically increases with increasing distance from isocentre).

The AAPM recommends a periodic test for the gradient non‐linear component (GNL) and specifies tolerances for two different volume sizes, indicated with the diameter spherical volume (DSV). The IPEM specifies two different tolerances for the ‘relevant volume’ and makes distinction between SRS and non‐SRS. The IPEM report does not make a distinction between the linear and non‐linear components. NCS has adopted a combination of the two approaches, where a ‘relevant volume’ is specified for smaller DSV (SRS applications), but fixed DSVs are specified for large FOV measurements. For diagnostic MRI the NCS recommends that a test of the linear (scaling) component suffices, whereas for applications (MR‐sim, MR‐only, and MR‐Linac) the non‐linear components should be tested as well. The minimum frequency is set annually or after equipment change by all three guidelines, but more regular checks recommended to test stability of the gradient amplitude calibration over time.

‐ For test 8, B_0_ homogeneity, the three reports compare very similarly, but subtle differences are observed in the sense that the IPEM report specifies the tolerances depending on the use case (SRS vs non‐SRS), whereas the NCS and AAPM report specify tolerances for different volume sizes. Note that the NCS defines the tolerance in parts‐per‐million (ppm) for MR‐sim and MR‐only, but in Hz for the MR‐Linac. The authors state that this was done to have a tolerance that,, scales with field strength and is therefore matched for 0.35T and 1.5T MR‐Linacs in absolute terms. Finally, it is worth pointing out that the AAPM and NCS tolerances are given in frequency offsets (either in ppm or in Hz), whereas the tolerance specified by the IPEM is in mm. This makes the IPEM test dependent on the readout bandwidth of the scan protocol. The minimum frequency is set to annually or equipment change by the IPEM and AAPM. The NCS recommends a more frequent, quarterly, frequency.

Overall, we see that the tolerances and recommended frequency are similar but not identical for the three guidelines. However, in practice these differences are relatively minor. In some cases, a preferable tolerance in one report is equal to the strict tolerance in another (with no preferable tolerance given). These tolerances are either a translation from earlier, established reports on CT‐sim and onboard Cone‐beam CT (CBCT), and consensus among the authors of each respective guideline. A slight variation between the three reports is therefore inevitable and provides some additional insight into how critical the tolerances are. Ultimately the Medical Physics Expert should decide which tolerances to use based on all the other sources of error and the treatment margins of a specific application in mind.

In addition to the QA tolerances summarized here, it is important to note that all three guidelines include dedicated sections on *protocol optimisation for RT*, in which the selection of sequence parameters is discussed in the context of both image quality and geometric fidelity (see Table 1). These sections explicitly address the inherent trade‐offs between parameters that reduce geometric distortions (e.g., increased bandwidth or the application of gradient non‐linearity correction) and their impact on image quality metrics such as SNR and image resolution.

## 4 CONCLUDING REMARKS

More than two decades after the first scientific papers on MRI in RT, three key international guidance reports have been published on the integration of MRI in this field. This editorial provides a comparative analysis of the reports from the IPEM, AAPM, and NCS to assist Medical Physics Experts in determining which source to consult for specific information. Our analysis concludes that the recommendations across all three reports are largely consistent and can be used in a complementary manner depending on use case.

## CONFLICT OF INTEREST STATEMENT

R.H.N. Tijssen reports research collaboration with Modus Medical Inc. S.F. Petit has no conflicts of interest to disclose. M. de Smet has no conflicts of interest to disclose. Z. van Kesteren has no conflicts of interest to disclose. C. Glide‐Hurst reports research collaborations with Modus Medical Inc, RaySearch, Leo Cancer Care, GE Healthcare, and Medscint Inc outside the submitted work. E. Paulson reports research support from Siemens Healthineers and Philips Healthcare. R. Speight has no conflicts of interest to disclose. M.J. Dubec has no conflicts of interest to disclose. E.J.L. Brunenberg has no conflicts of interest to disclose. J.P.A. Kuijer has no conflicts of interest to disclose.
